# Women's knowledge and attitudes regarding alcohol consumption in pregnancy: a national survey

**DOI:** 10.1186/1471-2458-10-510

**Published:** 2010-08-23

**Authors:** Elizabeth Peadon, Jan Payne, Nadine Henley, Heather D'Antoine, Anne Bartu, Colleen O'Leary, Carol Bower, Elizabeth J Elliott

**Affiliations:** 1 Discipline of Paediatrics and Child Health, Sydney Medical School, The University of Sydney, The Children's Hospital at Westmead, Locked Bag 4001, Westmead, NSW 2145 Australia; 2 Telethon Institute for Child Health Research, Centre for Child Health Research, The University of Western Australia, PO Box 855, West Perth Western Australia 6872 Australia; 3 Centre for Applied Social Marketing and Research, Edith Cowan University, 270 Joondalup Drive, Joondalup Western Australia 6027 Australia; 4 School of Nursing and Midwifery, Faculty Health Sciences, Curtin University of Technology, GPO Box U1987 Perth Western Australia 6845 Australia

## Abstract

**Background:**

Alcohol exposure in pregnancy is a common and modifiable risk factor for poor pregnancy and child outcomes. Alcohol exposure in pregnancy can cause a range of physical and neurodevelopmental problems in the child including the Fetal Alcohol Spectrum Disorders (FASD). In order to improve prevention strategies, we sought to describe the knowledge and attitudes of women of childbearing age regarding alcohol consumption during pregnancy and its effects on the fetus.

**Methods:**

We conducted a national cross-sectional survey via computer assisted telephone interview of 1103 Australian women aged 18 to 45 years. Participants were randomly selected from the Electronic White Pages. Pregnant women were not eligible to participate. Quotas were set for age groups and a minimum of 100 participants per state to ensure a national sample reflecting the population. The questionnaire was based on a Health Canada survey with additional questions constructed by the investigators. Descriptive statistics were calculated and logistic regression analyses were used to assess associations with participants' knowledge and attitudes.

**Results:**

Of women surveyed, 61.5% had heard about effects of alcohol on the fetus and 55.3% had heard of Fetal Alcohol Syndrome. Although 92.7% agreed alcohol can affect the unborn child, 16.2% did not agree that the disabilities could be lifelong. Most women agreed that pregnant women should not drink alcohol (80.2%) and 79.2% reported having negative feelings towards pregnant women drinking alcohol. Women with higher education levels were more likely to know the effects of alcohol consumption in pregnancy (adjusted OR 5.62; 95% CI 3.20 to 9.87) but education level and knowledge were not associated with attitude.

**Conclusions:**

There was a disjunction between knowledge and attitudes towards alcohol consumption in pregnancy. These findings will assist in developing effective health promotion campaigns to reduce fetal alcohol exposure and subsequent fetal damage.

## Background

Alcohol exposure is one of the few modifiable risk factors for poor pregnancy outcomes. Fetal Alcohol Syndrome (FAS) was formally described only thirty-five years ago[1] but the effects of alcohol consumption in pregnancy on the unborn child have been recognised for hundreds of years[2]. Alcohol consumption in pregnancy has been associated with miscarriage[3], premature birth[4], stillbirth[5], low birth weight[5] and diagnoses that are encompassed by the umbrella term of the Fetal Alcohol Spectrum Disorders (FASD). Children with FAS have characteristic facial features (small palpebral fissures, smooth philtrum and thin vermillion border of the upper lip), prenatal and/or postnatal growth retardation, and central nervous system structural and/or functional abnormalities. Partial FAS, Alcohol Related Neurodevelopmental Disorder (ARND) and Alcohol Related Birth Defects (ARBD) are other diagnoses covered by FASD[6].

Alcohol consumption is common in many cultures and consumption in young women has increased over the past thirty years, with an increasingly frequent pattern of risky drinking and increased risk of unintentional alcohol consumption during pregnancy[7-9]. Up to 50% of pregnancies are unplanned[10] and there is an association between binge drinking and unplanned conception[8,9]. Consequently, there is potentially a high rate of fetal exposure to alcohol.

We have estimates of alcohol consumption in pregnancy[10], however we have little contemporary data about women's knowledge and attitudes regarding alcohol consumption in pregnancy and how these are associated with alcohol consumption in pregnancy. In order to design effective health promotion strategies to reduce alcohol consumption in pregnancy, we need to understand potential influences on women's behaviour. Our aims in this study were to ascertain women's knowledge and attitudes regarding alcohol consumption in pregnancy and its effects on the unborn child and to identify maternal factors associated with knowledge and attitudes.

## Methods

### Participants and Sampling

Random sampling was achieved by computer-generated national sampling from the Electronic White Pages. Women aged 18 to 45 years inclusive were eligible to participate in the survey. Pregnant women were deemed ineligible to participate to comply with the Ethics Committee's recommendation that the topic might generate anxiety. Standard questions regarding age, gender and pregnancy status were used to screen participants' eligibility. The survey was administered in English. To ensure that the survey population was a representative national sample, we stratified the sample by age group (18 to 29 years and 30 to 45 years) and state of residency. We set quotas to reflect the age distribution of Australian women in the 18 to 45 year age group in 2003[11], and set a minimum of 100 participants from each state. The survey was administered as a computer assisted telephone interview by the Survey Research Centre, School of Population Health, University of Western Australia in August and September, 2006.

### Questionnaire

Our "Alcohol and Pregnancy Questionnaire" was based on the Health Canada survey "Alcohol Use During Pregnancy and Awareness of Fetal Alcohol Syndrome"[12] but the language was modified for the Australian context, with additional questions on women's attitudes to alcohol consumption in pregnancy (relating to cognitions and feelings). Decisions about changes to wording of the Health Canada survey questions and the construction of additional questions were made by investigator consensus. A pilot study of 20 randomly selected women was conducted in August 2006. Feedback from the pilot sample was reviewed by the investigators and was used to inform minor changes made to the questionnaire. The changes were clarification of instructions to the survey administrators and changes to recording and coding of responses for some questions. We collected demographic data (age, ethnicity, parity, postcode, educational level, employment, marital status) and data on women's knowledge and attitudes regarding alcohol consumption in pregnancy; and their knowledge of the effects of alcohol on the fetus.

### Statistical analysis

A sample size of n = 1100 provided estimates within 2% for variables with a prevalence of 40 to 50%. Data were analysed using SPSS (version 15.0, SPSS Chicago, IL, USA). Descriptive statistics of responses to survey questions were calculated. Associations between demographic characteristics and the survey outcomes, knowledge and attitudes regarding alcohol consumption in pregnancy were investigated, as well as the association between knowledge and attitude. Forward stepwise logistic regression analysis was conducted to analyse which factors were associated with knowledge and attitudes. Odds ratios and 95% confidence intervals (CI) were calculated. These analyses included: age, educational attainment, pregnancy history, smoking status, alcohol consumption status, relationship status, country of birth (Australia versus overseas), and employment status. Dichotomous variables were created for age (18 to 29 years versus 30 to 45 years), pregnancy history (previously given birth versus nulliparous), smoking status (current smoker versus non or ex-smoker), alcohol consumption status (drank alcohol in the last 12 months versus no alcohol in the past 12 months), relationship status (de facto partner or married versus single), and employment status (employed versus not employed). Educational attainment responses were categorized as completed less than Year 12, completed Year 12, completed a post-school qualification lower than a bachelor degree and completed a bachelor degree or higher. Knowledge responses were obtained from yes/no questions or agreement on a 5-point Likert scale. The responses on the Likert scale were dichotomised (agree versus not agree). Participants' attitudes to the scenario of seeing a pregnant woman drinking were dichotomised to negative versus neutral or positive. A priori, age, educational attainment and pregnancy history were considered potential confounders and all associations were adjusted for these factors.

### Ethics

The study protocol was approved by the Ethics Committees of the Women's and Children's Health Service, Perth, Western Australia, The University of Sydney and the Western Australian Aboriginal Health Information and Ethics Committee.

## Results

Of 1603 eligible women contacted, 1103 (68.8%) consented and completed the interview. The demographic characteristics of respondents were similar to those of women in the general population, except that respondents were more likely to have been born in Australia, had a pregnancy, and achieved a higher level of education (Table 1) [13-15].

**Table 1:** Characteristics of survey respondents (n = 1103)

Characteristic	n	(%)	Australian Population%
*Age *			
Age, years, mean, (standard deviation)	32.0	(8.3)	
18-29 years	439	(39.8)	39.8[13]
30-45 years	664	(60.2)	60.2[13]
			
*Pregnancy History*			
Given birth to a child	700	(63.5)	57.8[13]
Years since last child born, median (range)	5.0	(<1 to 23)	
Planning pregnancy in the next 2 years	230	(20.9)	
Planning pregnancy sometime in the future (n = 873)	274	(31.4)	
			
*Marital status*			
Married (or de facto/life partner)	697	(63.2)	61.2[13]
Never married	329	(29.8)	27.7[13]
Divorced	43	(3.9)	6.9[13]
Separated	29	(2.6)	3.6[13]
Widowed	5	(0.5)	0.6[13]
			
Aboriginal or Torres Strait Islander origin	21	(1.9)	2.4[13]
			
*Country of Birth*			
Australia	876	(79.4)	70.4[13]
Years in Australia if born outside Australia, median (range)	18	(<1 to 44)	
			
*Current employment status (n = 1102)*			
Currently employed	702	(63.6)	67.9[13]
Home duties	230	(20.9)	
Student	127	(11.5)	
Unemployed or retired or on a pension	43	(3.8)	4.1[13]
			
*Highest educational attainment*			
Year 11 or below	174	(15.8)	30.6[14]
Year 12	252	(22.8)	22.2[14]
Post-school qualification below bachelor degree	383	(34.7)	22.6[14]
Bachelor degree	225	(20.4)	16.4[14]
Postgraduate qualification	69	(6.3)	7.6[14]
			
*Behaviour*			
Drank alcohol in the past 12 months	986	(89.4)	84.2[15]
Smoke cigarettes	199	(18.0)	17.7[15]

### Knowledge about alcohol and pregnancy

When asked 'Have you ever heard of any effects on pregnancy or the unborn child which are caused by drinking alcohol during pregnancy, 61.5% of respondents said 'yes' (Table 2). The respondents who said "yes" (n = 678) were asked "can you tell me what are the effects on the pregnancy or unborn child of drinking alcohol during pregnancy?" Women were allowed to nominate more than one effect. The most common effects nominated were Fetal Alcohol Syndrome (31.7%), low birth weight (28.5%) and brain damage (15.6%) (Table 2). For the purpose of further analysis, the responses were categorized into types of effect of alcohol consumption in pregnancy. Overall, 36.7% of respondents nominated neurobehavioural effects (delayed development, low IQ, learning disabilities, brain damage, mental disorders, behavioural problems and Attention Deficit Disorder); one third (33.5%) nominated one or more of the diagnoses resulting from alcohol exposure in pregnancy (Fetal Alcohol Syndrome, Fetal Alcohol Spectrum Disorder, Fetal Alcohol Effects, Alcohol Related Birth Defects and Alcohol Related Neurodevelopmental Disorders); and 33.9% nominated growth problems (low birth weight or growth problems). (Data not shown)

**Table 2:** Knowledge of the effects on the unborn child of alcohol consumption during pregnancy

Knowledge	n(%)
Have ever heard of any effects on pregnancy or the unborn child which are caused by drinking alcohol during pregnancy (n = 1103)	678 (61.5)
	
Nominated effects of alcohol on pregnancy or the unborn child (n = 678)*	
Fetal Alcohol Syndrome	215 (31.7)
Low birth weight	193 (28.5)
Brain damage	106 (15.6)
Baby addicted/experiences withdrawal	99 (14.6)
Causes birth defects/deformities	74 (10.9)
Mental disorders	61 (9.0)
Premature birth	59 (8.7)
Delayed development	58 (8.6)
Growth problems	52 (7.7)
Physical disorders	46 (6.8)
Learning disabilities	44 (6.5)
Harmful/ill effects	44 (6.5)
Don't know	40 (5.0)
Lower IQ	24 (3.5)
Cranial/facial deformities	20 (2.9)
Baby born with alcohol in its system	13 (1.9)
Attention Deficit Disorder	11 (1.6)
Behavioural Problems	10 (1.5)
Miscarriage	10 (1.5)
Alcohol Related Neurodevelopmental Disorder	7 (0.9)
Alcohol Related Birth Defects	6 (0.9)
Fetal Alcohol Spectrum Disorders	5 (0.7)
Fetal Alcohol Effects	4 (0.6)
Stillbirth	2 (0.3)
Other	40 (5.9)
	
Have ever heard of (n = 1103)	
Fetal Alcohol Syndrome	610 (55.3)
Alcohol Related Birth Defects	486 (44.1)
Alcohol Related Neurodevelopmental Disorder	194 (17.6)
Fetal Alcohol Spectrum Disorders	94 (8.5)

*Women volunteered this information and nominated one or more effects

The respondents were asked if they had heard of specific disorders associated with alcohol exposure in pregnancy. The proportion of respondents who said "yes" varied from 55.3% for Fetal Alcohol Syndrome to 8.5% for Fetal Alcohol Spectrum Disorder (Table 2). Respondents who had heard of a disorder were asked to describe the characteristics of the disorder. Knowledge of the characteristics of these conditions was poor. "Don't know" was the most or second most common response when a respondent who had heard of a condition was asked to describe the disorder, ranging from 55.3% for FASD to 21.8% for FAS. Incorrect or non-specific responses such as "Effects of alcohol on fetus" were also common.

### Attitudes about alcohol consumption in pregnancy

Attitudes about alcohol consumption in pregnancy were assessed with a series of questions relating to cognitions and feelings. Respondents were asked to rate their agreement with statements about alcohol and pregnancy, some including an element of approval/disapproval, on a five-point Likert scale ranging from strongly agree to strongly disagree. Most respondents (80.2%) agreed that pregnant women should not drink alcohol; 37.2% agreed and 43.1% strongly agreed with this statement. Most (92.7%) agreed that drinking alcohol during pregnancy can affect the unborn child and that the more alcohol a woman drinks, the more likely the unborn child will be harmed (96.6%); however 16.2% did not agree that drinking alcohol during pregnancy can lead to lifelong disabilities in a child. Only 67.5% of respondents agreed that women are aware of the effects that drinking alcohol during pregnancy can have on the unborn child. More than three quarters of respondents (78.8%) agreed that members of the community are concerned about women drinking alcohol during pregnancy. Most women (91.9%) agreed that pregnant women should drink less than seven standard alcohol drinks each week, and only a minority agreed that it is ok for pregnant women to drink three or four standard alcohol drinks on one day (2.1%) and that it is ok for pregnant women to become intoxicated (0.8%).

**Figure 1:**
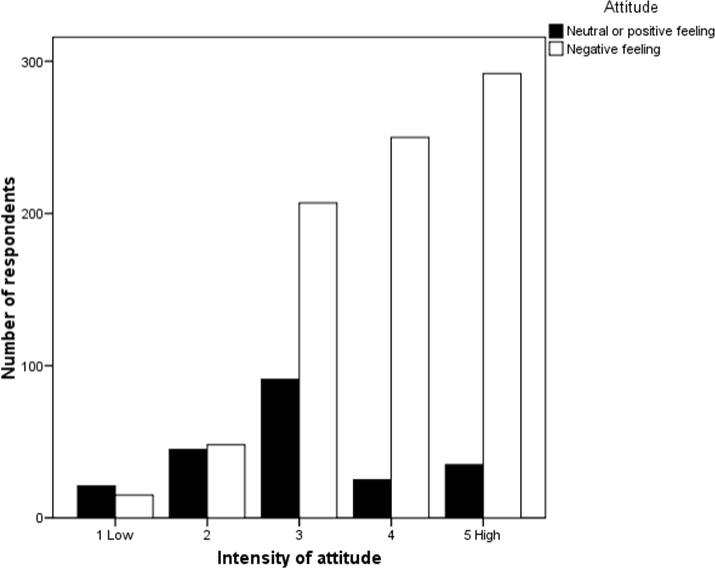
**Intensity of attitudes towards a pregnant woman drinking alcohol (n = 1103)**.

When respondents were asked how they would feel if they saw a pregnant woman drinking alcohol, the majority (79.2%) reported negative attitudes including "concerned", "disappointed", "upset" or "angry". The remaining 20.8% reported neutral or positive attitudes such as "OK", "comfortable" or "not bothered". Respondents were asked to rate the intensity of their feeling on a scale of one (low) to five (high) (Figure 1). Negative attitudes were rated significantly higher in intensity than neutral or positive attitudes (p < 0.001).

### Factors associated with knowledge of the effects on the unborn child of alcohol consumption in pregnancy (Table 3)

**Table 3:** Factors associated with knowledge of the specific effects on the child of alcohol exposure in pregnancy

	Know one or more of the diagnoses resulting from alcohol exposure in pregnancy	Know that alcohol causes growth problems	Agree that drinking alcohol during pregnancy can lead to lifelong disabilities in a child	Agree that pregnant women should not drink alcohol	Report negative feeling to women drinking alcohol in pregnancy
	Adjusted OR^† ^(95% CI)				
Highest education level					
<Year 12	Reference	Reference	Reference	Reference	Reference
Completed Year 12	1.87* (1.01 to 3.46)	0.98 (0.56 to 1.70)	1.13 (0.68 to 1.89)	1.08 (0.64 to 1.81)	0.80 (0.48 to 1.34)
Post school below bachelor degree	1.48 (0.83 to 2.65)	1.52 (0.95 to 2.44)	1.32 (0.85 to 2.07)	0.88 (0.56 to 1.38)	0.89 (0.57 to 1.40)
Bachelor degree or higher	5.62** (3.20 to 9.87)	2.04* (1.26 to 3.31)	2.15* (1.28 to 3.61)	0.87 (0.54 to 1.40)	0.75 (1.47 to 1.21)
Alcohol can cause lifelong disabilities				4.59** (3.22 to 6.54)	3.67** (2.56 to 5.29)
Pregnancy history					
Not given birth to a child	Reference	Reference	Reference	Reference	Reference
Given birth to a child	1.13 (0.77 to 1.67)	1.24 (0.84 to 1.83)	0.64 (0.40 to 1.01)	0.59* (0.39 to 0.88)	0.39** (0.26 to 0.60)
Smoker				0.48** (0.34 to 0.69)	0.78 (0.53 to 1.14)
Age					
18 to 29 years	Reference	Reference	Reference	Reference	Reference
30 to 45 years	0.72 (0.49 to 1.06)	1.18 (0.81 to 1.72)	0.67 (0.43 to 1.03)	1.01 (0.69 to 1.48)	1.14 (0.77 to 1.68)

^† ^Odds ratios for each variable adjusted for age, highest education level and pregnancy history

*p < 0.05. **p < 0.001.

Knowledge was related to education level. Women who had a bachelor degree or higher were more likely than those who had left school before the end of year 12 to know that alcohol consumption in pregnancy can cause one or more of the diagnoses resulting from alcohol exposure in pregnancy (OR 5.60 95% CI 3.22 to 9.74; p < 0.001), and to know that drinking alcohol during pregnancy can lead to lifelong disabilities in a child (OR 2.52 95% CI 1.52 to 4.19; p < 0.001). Women who had previously given birth were less likely to know that the disabilities can be lifelong than nulliparous women (OR 0.48 95% CI 0.33 to 0.69; p < 0.001). Similarly, women aged 30-45 years were less likely than younger women to know that alcohol can cause growth problems or that it can cause lifelong disabilities. After adjusting for potential confounders, level of education remained the only statistically significant predictor of women's knowledge.

### Factors associated with attitudes to alcohol consumption in pregnancy (Table 3)

Agreeing that alcohol causes lifelong disabilities, planning a future pregnancy, age, pregnancy history and smoking status were associated with attitudes to alcohol consumption during pregnancy. After adjusting for age, level of education and pregnancy history, women who agreed that alcohol consumption in pregnancy can cause lifelong disabilities were more likely than women without this knowledge to agree that pregnant women should not drink (adjusted OR 4.59 95% CI 3.22 to 6.54; p < 0.001) and to have a negative attitude towards alcohol consumption in pregnancy (adjusted OR 3.67 95% CI 2.56 to 5.29; p < 0.001). This was the only significant association between knowledge and attitude. Women who had given birth previously or were smokers were more likely to have a tolerant attitude towards alcohol consumption in pregnancy. Education level was not associated with attitudes to alcohol consumption in pregnancy.

## Discussion

This survey provides contemporary national data on Australian women's knowledge and attitudes about alcohol consumption in pregnancy and its effects on the fetus. While there was an encouraging baseline level of knowledge regarding alcohol use in pregnancy and its effects on the fetus, it is of concern that one in three women of child bearing age said they did not know any adverse effects of alcohol consumption in pregnancy. Many women who said they were aware of adverse effects could not name any specific effects. Most women agreed that pregnant women should not drink alcohol and reported a negative attitude towards alcohol consumption in pregnancy, although one fifth had a neutral or positive attitude towards alcohol consumption in pregnancy.

Comparison of results of this survey with those from the 2006 Canadian survey[16] showed some differences in knowledge. Australian women were less likely than Canadian women (83.8% vs 96%) to agree that alcohol consumption in pregnancy can lead to life long disabilities. Fewer Australian than Canadian women had heard of the terms FAS or FASD (56.4% vs 88%). Australian women were also more likely to report they did not know any specific characteristics of these disorders (25.7% vs 18%). This is the third iteration of the Canadian survey and there has been an increase in knowledge since the first survey in 1999, which may be the outcome of several FASD awareness campaigns conducted in Canada[17].

Direct comparison to other studies is hampered by the use of different questions; however, women's knowledge in this study was generally similar to that in other studies. In a French study, 83% of respondents knew that alcohol in pregnancy could be harmful to the baby[18]. In a Danish study, 85% of respondents knew that alcohol is possibly harmful to the fetus. However, a much smaller proportion of Danish women stated that pregnant women should not drink alcohol than in this study (80.2% vs 24%). This may be due to differences in methodology, as the Danish women gave a spontaneous answer to an open-ended question, whereas the women in this study were asked to agree or disagree with a statement[19].

Women with higher levels of education had more knowledge about the specific effects of alcohol consumption in pregnancy, which has been found in previous studies[16]. However, women's attitudes towards alcohol consumption in pregnancy were not associated with level of education (the Canadian survey did not report women's attitudes). Agreeing that alcohol in pregnancy can cause lifelong disabilities in the child was the only knowledge associated with a negative attitude.

Knowledge, experiences, social influence, habits, self confidence, motivation, attitudes and possibility for change have all been identified as determinants of health behaviour. One common theory is that knowledge is required to influence attitude which then leads to changes in health behaviour. However, other models suggest that there is a more complex pathway leading to changes in health behaviour, e.g. experiences and social influence may cause changes in self-confidence and attitude which lead to changes in health behaviour[20]. Other studies have also found a disjunction between knowledge and attitudes, which may be mediated by risk perception[19,21]. Risk perception, which is strongly influenced by individual experience, is an important predictor of alcohol consumption during pregnancy[21,22]. Differences in risk perception may explain the differences in attitudes between nulliparous and multiparous women. Women who have had a previously health pregnancy report a lower perceived risk from alcohol consumption than women experiencing their first pregnancy[21].

Traditional public health approaches have concentrated on increasing public knowledge through awareness campaigns[17,21]. However, it is clear that approaches to the prevention of FASD must encompass more than education. Awareness campaigns in isolation are doomed to failure as they do not address attitudes, social influence or experience. The data from this study can be used to inform development of effective health promotion campaigns and identify groups who require targeted strategies. These findings suggest we need a combination of strategies at community and society levels to inform Australian women's knowledge and influence attitudes about alcohol and pregnancy. At the community level, strategies could include education in schools and social marketing campaigns. Prevention of tobacco smoking campaigns may provide successful models which can be adapted for this issue. At the society level, governments should disseminate clear guidelines about alcohol consumption in pregnancy to health professionals and provide funding for public health campaigns. Other government strategies for raising awareness include legislation for compulsory labelling of alcoholic beverages.

This is the first national Australian study reporting women's knowledge and attitudes towards alcohol consumption in pregnancy and the first study to explore the relationship between knowledge and attitudes. The study has several strengths: the response rate was high (nearly 70% of eligible women contacted agreed to participate) and the study population was similar to the Australian population of women aged 18 to 45 years, although with a higher level of education. As knowledge of the effects of alcohol consumption in pregnancy is associated with level of education, our estimate of the level of knowledge in the general population may be inflated. A limitation of the study is that it was not specifically designed to capture knowledge and attitudes of Indigenous women, who comprised 1.9% of our sample, slightly lower than the proportion in the general population (2.4%). The knowledge and attitudes regarding alcohol and pregnancy may be different in Indigenous women compared to non-indigenous women and a separate study of Indigenous women's knowledge and attitudes is warranted. Also, teenagers under the age of 18 years were omitted from this study. There were 11, 977 births to Australian teenagers in 2006[23]. As there has been an increase in the proportion of teenagers drinking at harmful levels since 1990[24], contemporary research into their knowledge and attitudes towards alcohol consumption in pregnancy is needed.

## Conclusions

In this study, we found that Australian women of childbearing age have poor knowledge of the specific effects of alcohol in pregnancy on the unborn child and one in five women have a neutral or positive attitude towards alcohol consumption in pregnancy. We also found a disjunction between women's knowledge and attitudes. Interventions based on a theoretical model of health behaviour and which address past experience, social influence, risk perception and identified gaps in knowledge and misconceptions may be more successful than the traditional educational approaches[21]. Public health campaigns, national policy and guidelines must all be underpinned by a regularly reviewed scientific evidence base of the influences on women's alcohol behaviour in pregnancy and effective prevention strategies.

## Competing interests

The authors declare that they have no competing interests.

## Authors' contributions

EP, JP, NH, HD, AB, AB, CO, CB and EJE conceived and designed the study; EP and JP conducted the analysis; EP wrote the first draft of the paper. All authors contributed to writing the paper and approved the final version for publication. EP is guarantor for the study.

## Pre-publication history

The pre-publication history for this paper can be accessed here:

http://www.biomedcentral.com/1471-2458/10/510/prepub
